# Re-revision following revision of a failed primary reverse total shoulder arthroplasty: an analysis of the National Joint Registry and Hospital Episode Statistics for England

**DOI:** 10.1016/j.jseint.2025.04.030

**Published:** 2025-05-22

**Authors:** Olivia O'Malley, Andrew Davies, Amar Rangan, Sanjeeve Sabharwal, Peter Reilly

**Affiliations:** a Department of Bioengineering, Cutrale Perioperative & Ageing Group, Imperial College London, London, UK; b Department of Trauma & Orthopaedics, Imperial College Healthcare NHS Foundation Trust, London, UK; c Hull York Medical School & Department of Health Sciences, University of York, York, UK

**Keywords:** Reverse shoulder arthroplasty, Revision reverse shoulder arthroplasty, Re-revision shoulder arthroplasty, Reverse shoulder replacement, Revision shoulder replacement, Shoulder instability

## Abstract

**Background:**

Reverse total shoulder arthroplasty (rTSA) is the most common type of shoulder replacement in the UK and its use continues to rise. There is minimal data in the literature looking at re-revision following revision of a failed rTSA. This study utilizes the National Joint Registry and Hospital Episode Statistics for England to calculate the incidence and risk factors for re-revision of a failed primary rTSA.

**Methods:**

Patients were included if they had a revision procedure between April 1, 2012, and March 31, 2022. This National Joint Registry database was linked to Hospital Episode Statistics and Office of Population Censuses and Surveys Classification of Interventions and Procedures version 4.9 codes were used to identify a further revision procedure. The primary outcome was implant survival, the Kaplan-Meir method was used for analysis. Secondary outcomes were risk factors that predispose a patient to a further revision procedure. A multivariable regression analysis was performed to assess for independent risk factors for re-revision.

**Results:**

There were 685 patients who underwent a revision of a primary rTSA done by 244 consultant surgeons with a median caseload of 4 patients (interquartile range 2-7) over a 10-year period. At 1 year, the incidence of re-revision was 15.91%; at 3 years, it was 21.41%; and at 5 years, it was 23.18%. A 1-year decrease in age resulted in a 4% increased risk of re-revision (hazard ratio 0.96 [95% confidence interval 0.94-0.98]), and if the primary revision was due to instability/dislocation, there was a 2-fold increased risk of re-revision compared to if the primary revision was done for any other indication (hazard ratio 2.47 [95% confidence interval 1.59-3.82]).

**Conclusion:**

Re-revision rates following revision rTSA are high with independent risk factors being younger age and instability as primary revision diagnosis. Given the risk profile and low volume of revision cases performed by surgeons, centralizing revision rTSA surgeries to high-volume centers may warrant further exploration to improve outcomes.

Reverse total shoulder arthroplasty (rTSA) was originally designed for cuff-tear arthropathy in a demographic that was generally older requiring less longevity of the implant. Despite this, rTSA is now being used for a much broader range of indications such as primary osteoarthritis, acute trauma, trauma sequalae, and cuff tears without arthropathy. It is now the most widely used type of shoulder arthroplasty in the UK, the USA, and Australia.^2^^,^^4^^,^^15^ The current reported 5-year revision rate of rTSA in the UK is 3.05%; it is expected, however, that this will rise as the use of rTSA expands in terms of indication but also patient demographic.^14^^,^^15^^,^^21^ Despite this rapid increase in use of rTSA and the expected increased revision burden, there is little evidence in the literature regarding the outcomes of patients who go on to have a revision. A recent systematic review estimated a high re-revision rate of approximately 14% at 1 year and 23% at 5 years; however, due to the lack of high quality evidence, a survival analysis was unable to be performed.^17^ There is also limited literature regarding predictors of failure when an rTSA is revised. One recent study looked at predictors of revision in rTSA, which found younger age, male sex, and nonosteoarthritis indication for primary as independent predictors of revision; however, predictors of failure following revision were not specifically reviewed.^3^ This study aims to utilize the National Joint Registry (NJR) and Hospital Episode Statistics (HES) to calculate the incidence of re-revision following revision of a primary rTSA and identify risk factors for failure of primary revision.

## Methods

### Data source

Data were requested from the NJR for all patients receiving an rTSA, and patients were included in the study if they had an outcome of ‘Revised.’ Revision took place between the April 1, 2012, and March 31, 2022. The NJR implemented mandatory data collection of shoulder replacements since 2012 and regularly audits its completeness in order to ensure compliance in reporting. In the 2018/19 audit cycle, data were 96.92% complete for primary replacements and 97.67% for revision replacements.^8^^,^^15^ Data that were missing were deemed to be missing at random and should not bias any results or conclusions from analyzing the registry data.^15^ This database was then linked to the HES database using NJR index number. HES is a database containing information about admissions, accident and emergency attendance, and outpatient appointments at NHS Hospitals in England. HES data cover all NHS Clinical Commissioning Groups including private patients using NHS services, patient's resident outside England, and care delivered in treatment centers funded by the NHS. HES data are used to reimburse hospitals for the care they provide by NHS England and undergoes internal data quality checks as well as automated data cleaning to ensure good internal validity.^19^ Indication for Primary Procedure was identified using the NJR database. A total of 11.97% had multiple indications; these were sorted into mutually exclusive groups where possible. A full explanation of this is detailed in Supplementary Appendix S1. The revision type and procedure coding on the shoulder data collection form by the NJR have varied over the study period (Minimum Data Set version 5-7). Therefore, procedure codes and types were cross referenced with implant data and indications for revision in order to create 6 categories: 1) single-stage revision, 2) two-stage revision, 3) debridement and implant retention (DAIR) ± modular exchange for infection, 4) modular exchange for indications other than infection, 5) excision arthroplasty, or 6) conversion to arthrodesis. The revision implant for those who had a single or two-stage revision was cross checked against listed implants in the NJR database to ensure correct coding of revision implants. The NJR records only first revision episode and therefore often the final implant data in those who had a two-stage revision was missing, using Office of Population Censuses and Surveys Classification of Interventions and Procedures version 4.9 revision codes final revision implants were identified from the HES database (Supplementary Appendix S2). The implants were coded as rTSA, hemiarthroplasty, or unspecified if the HES coding was unspecific to a single implant or data in the NJR was too limited to identify the implant. The implant was coded as missing if there was no implant data in the HES or NJR file. Indication for revision is recorded in the NJR file; however, revisions can be recorded for multiple indications. A hierarchy of indications was used to determine primary indication (Supplementary Appendix S3). A re-revision was identified using Office of Population Censuses and Surveys Classification of Interventions and Procedures version 4.9 codes in the HES data and the corresponding operation date recorded (Supplementary Appendix S2). For comorbidities to calculate Charlson Comorbidity Index (CCI) and review individual comorbidities, International Classification of Diseases 10th Revision codes were detected in the HES data at or before the primary revision episode (Supplementary Appendix S4).

### Outcomes

The primary outcome was re-revision at 1, 3, 5, 7, and 9 years. A subgroup analysis of re-revision with those patients who had a primary revision for infection excluded was performed. Secondary outcomes were the risk factors that predispose a patient to a re-revision procedure.

### Statistical analysis

The Kaplan-Meier method was used for implant survival. Demographic differences between the patients who did and did not receive a re-revision procedure were compared using Mann-Whitney U test for the skewed continuous variables, age, CCI, and surgeon volume, and a chi-squared test used for categorical variables. If >20% of the categorical variables had <5 patients, Fischer's exact test was used. For analysis of risk factors, a univariable cox regression analysis was done for each demographic variable to assess for predictors of re-revision. A multivariable cox regression analysis was then performed to assess for independent risk factors for re-revision. Demographic variables utilized for the univariable and multivariable analysis were selected based on previous literature that has reviewed predictors of revision or speculated on possible causes of failure of primary revision.^3^^,^^10^

## Results

**Figure 1:**
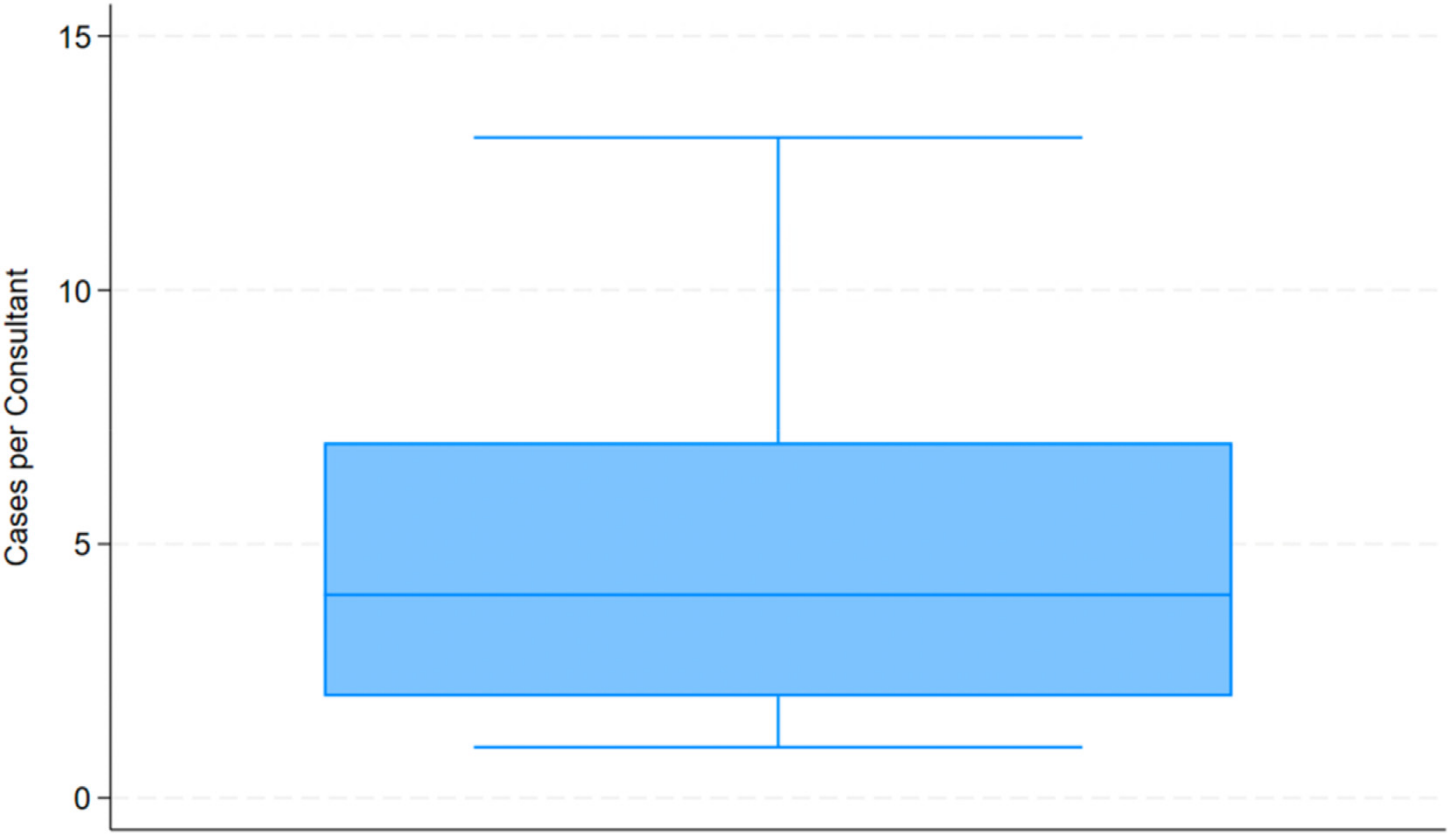
Box and whisker plot of revision case load by consultant.

There were 26,403 rTSA patients in the combined HES and NJR database with a mean follow-up of 4.90 years (standard deviation 2.41), and within this, 685 patients underwent revision between April 1, 2012, and March 31, 2022. These revisions were done by 244 consultant surgeons with a median caseload of 4 cases (interquartile range 2-7) over the 10-year period (Fig. 1). The demographics and revision procedures are presented in Table I.

**Table I:** Demographics of revised patients∗ not mutually exclusive.

Demographic	Revision cohort (n = 685)
Mean age at first revision (SD)	73.06 (9.52)
Gender	
Female (%)	340 (49.64)
Male (%)	345 (50.36)
Indication for primary (%∗)	
CTA	307 (44.82)
Primary OA	117 (17.08)
Trauma sequalae	121 (17.66)
Acute trauma	66 (9.64)
Cuff tear without arthropathy	14 (5.71)
Inflammatory arthropathy	31 (4.53)
AVN	22 (3.21)
OA normal cuff	17 (2.48)
Dislocation arthropathy	6 (2.45)
Mets and malignancy	2 (0.35)
Other	17 (2.48)
Indication for revision (%)	
Instability/dislocation	229 (33.43)
Infection	183 (26.72)
Aseptic loosening	82 (11.97)
Fracture	55 (8.03)
Component dissociation	27 (3.94)
Unexplained pain	7 (1.02)
Cuff insufficiency	7 (1.02)
Impingement	4 (0.58)
Glenoid implant wear	3 (0.04)
Lysis humerus	2 (0.29)
Lysis glenoid	1 (0.15)
Stiffness	1 (0.15)
Other/unspecified	84 (12.26)
Revision procedure (%)	
Single-stage revision	390 (56.93)
Two-stage revision	172 (25.11)
Modular exchange for indications other than infection	77 (11.24)
DAIR ± modular exchange for infection	35 (5.11)
Excision arthroplasty	10 (1.46)
Conversion to arthrodesis	1 (0.15)
Revision implant (%)	
Hemiarthroplasty	50 (7.30)
Reverse	423 (61.75)
Non specified/other	77 (11.24)
Missing implant data	12 (1.75)
Modular exchange	112 (16.35)
Excision arthroplasty	10 (1.46)
Arthrodesis	1 (0.15)
Comorbidities (%)	
Previous myocardial infarction	95 (13.87)
Congestive cardiac failure	69 (10.07)
Peripheral vascular disease	80 (11.68)
Cerebrovascular disease	118 (17.23)
Dementia	43 (6.28)
Chronic pulmonary disease	233 (34.01)
Rheumatic disease	142 (20.73)
Peptic ulcer disease	53 (7.74)
Mild liver disease	46 (6.72)
Diabetes without complications	150 (21.90)
Diabetes with end organ damage	30 (4.38)
Hemi or paraplegia	30 (4.38)
Moderate or severe renal disease	98 (14.31)
Malignancy except skin	104 (15.18)
Lymphoma	6 (0.88)
Leukaemia	9 (1.31)
Moderate or severe liver disease	13 (1.90)
Metastsis	27 (3.94)
HIV/AIDS	0 (0)
CCI median (IQR)	5 (3-6)

*IQR*, interquartile range; *DAIR*, débridement and implant retention; *CCI*, Charlson Comorbidity Index; *SD*, standard deviation; *HIV*, human immunodeficiency virus; *AIDS*, acquired immune deficiency syndrome; *CTA,* cuff tear arthropathy; *AVN,* avascular necrosis; *OA,* osteoarthritis.

∗ Not mutually exclusive.

**Figure 2:**
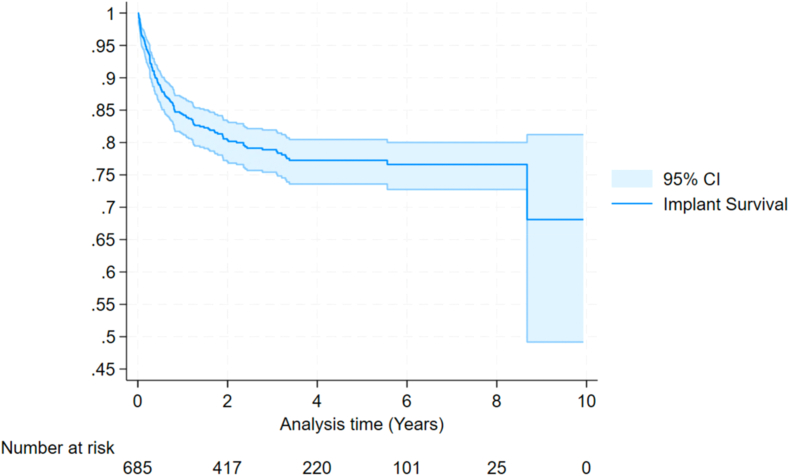
KM curve of re-revision. *CI*, confidence interval; *KM*, Kaplan Meir.

Of the 685 patients who underwent primary revision with a mean follow-up of 3.82 years (standard deviation 2.40), 139 (20.30%) went on to have a second revision. Figure 2 shows the Kaplan Meir curve for re-revision with cumulative revision rates in Table II.

**Table II:** Cumulative re-revision rates.

Cumulative revision by year % (95% CI)				
1 yr	3 yr	5 yr	7 yr	9 yr
15.91 (13.30-18.98)	21.41 (18.34-24.92)	23.18 (19.90-26.91)	23.79 (20.35-27.70)	28.71 (20.08-39.99)

*CI*, confidence interval.

**Figure 3:**
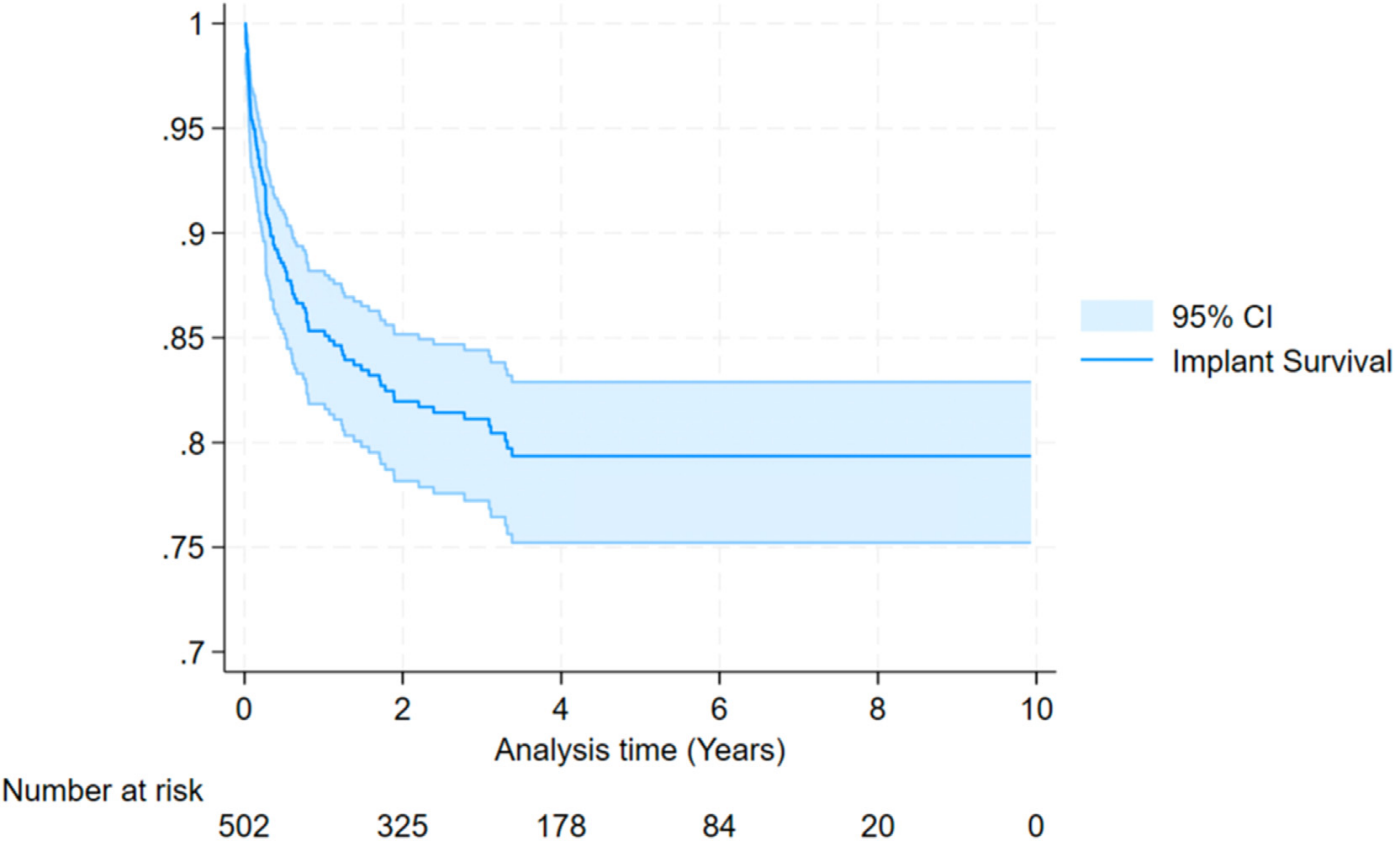
KM curve of re-revision in Aseptic Primary Revisions. *CI,* confidence interval; *KM,* Kaplan Meir.

The Kaplan Meir curve for re-revision in those who did not undergo their primary revision for infection is shown in Figure 3, and cumulative revision rates are presented in Table III.

**Table III:** Cumulative re-revision rates in aseptic primary revisions.

Cumulative revision by year % (95% CI)				
1 yr	3 yr	5 yr	7 yr	9 yr
14.96 (12.05-18.50)	19.15 (15.82-23.08)	21.04 (17.45-25.26)	21.04 (17.45-25.26)	21.04 (17.45-25.26)

Demographics of the patients that did and did not receive a re-revision were compared. This analysis found patients in the re-revision group were significantly more likely to be younger, male, and have a primary revision indication of instability/dislocation, and less likely to have a diagnosis of cerebrovascular disease, peptic ulcer disease, moderate or severe renal disease, malignancy, and have an overall lower CCI than those that did not require a further revision (Table IV). There was no difference between groups based on revision procedure, and there was no difference if the implant had been subsequently revised to a HA or rTSA.

**Table IV:** Demographic comparison of those rerevised and those with a successful primary revision.

Demographic	Re-revision cohort (n = 139)	No further revision (n = 546)	*P* value
Mean age at first revision (SD)	68.90 (10.63)	74.12 (8.92)	<.001
Mean age at re-revision (SD)	69.74 (10.57)	n/a	
Gender (%)			.02
Female	57 (41.01)	283 (51.83)	
Male	82 (58.99)	263 (48.17)	
Indication for first revision∗ (%)			
Instability/dislocation	59 (42.45)	170 (31.14)	.01
Infection	46 (33.09)	137 (25.09)	.06
Aseptic loosening	10 (7.19)	72 (13.19)	.05
Fracture	6 (4.32)	49 (8.97)	.07
Component dissociation	1 (0.72)	26 (4.76)	.03
Unexplained pain	0 (0)	7 (1.28)	.36
Cuff insufficiency	0 (0)	7 (1.28)	.36
Impingement	0 (0)	4 (0.73)	.59
Glenoid implant wear	0 (0)	3 (0.55)	1.00
Lysis humerus	0 (0)	2 (0.37)	1.00
Lysis glenoid	0 (0)	1 (0.18)	1.00
Stiffness	0 (0)	1 (0.18)	1.00
Other/unspecified	17 (12.23)	67 (12.27)	.99
Revision procedure (%)			.11
Single-stage revision	72 (51.80)	318 (58.24)	
Two-stage revision	48 (34.53)	13 (2.38)	
Modular exchange for indications other than infection	13 (9.35)	64 (11.72)	
DAIR ± modular exchange for infection	5 (3.60)	30 (5.49)	
Excision arthroplasty	1 (0.72)	9 (1.65)	
Conversion to arthrodesis	0 (0)	1 (0.18)	
Revision implant† (%)			.54
Hemiarthroplasty	9 (6.47)	41 (7.51)	
Reverse	88 (63.31)	335 (61.36)	
Comorbidities (%)			
Previous myocardial infarction	20 (14.39)	75 (13.74)	.84
Congestive cardiac failure	13 (9.35)	56 (10.26)	.75
Peripheral vascular disease	20 (14.39)	60 (10.99)	.27
Cerebrovascular disease	15 (10.79)	103 (18.86)	.02
Dementia	8 (5.76)	35 (6.41)	.78
Chronic pulmonary disease	50 (35.97)	183 (33.52)	.59
Rheumatic disease	29 (20.86)	113 (20.70)	.97
Peptic ulcer disease	4 (2.88)	49 (8.97)	.02
Mild liver disease	11 (7.91)	35 (6.41)	.53
Diabetes without complications	35 (25.18)	115 (21.06)	.30
Diabetes with end organ damage	8 (5.76)	22 (4.02)	.38
Hemi or paraplegia	7 (5.04)	19 (3.48)	.39
Moderate or severe renal disease	10 (7.19)	88 (16.12)	.01
Malignancy except skin	10 (7.19)	94 (17.22)	<.001
Lymphoma	1 (0.72)	5 (0.92)	.82
Leukaemia	2 (1.44)	7 (1.28)	.89
Moderate or severe liver disease	4 (2.88)	9 (1.65)	.34
Metastsis	3 (2.16)	24 (4.40)	.23
HIV/AIDS	0 (0)	0 (0)	1
CCI median (IQR)	4 (3-6)	5 (3-7)	<.001
Surgeon revision frequency	5 (2-9)	4 (2-7)	.01

*IQR*, interquartile range; *DAIR*, débridement and implant retention; *CCI*, Charlson Comorbidity Index; *SD*, standard deviation; *HIV*, human immunodeficiency virus; *AIDS*, acquired immune deficiency syndrome.

∗ Not mutually exclusive.

† Patients that had single/two-stage revision where an implant could be identified.

On univariable analysis age, male gender, indication for primary revision of instability, and infection, a two-stage revision procedure and a higher volume or revisions done by the primary surgeon were associated with an increased revision risk. Having a diagnosis of cerebrovascular disease, peptic ulcer disease, moderate or severe liver disease, and malignancy was associated with a decreased risk of having a revision procedure. On multivariable analysis, a 1-year reduction in age resulted in a 4% increased risk in requiring a re-revision operation and re-revision, a primary revision diagnosis of instability resulted in an over 2-fold increased risk of requiring a re-revision, and an increasing surgeon volume also conferred a 5% increase in re-revision risk per 1 case increase. A diagnosis of peptic ulcer disease, moderate or severe renal disease, and malignancy reduced the risk of requiring a revision operation (Table V).

**Table V:** Univariable and multivariable regression analysis of risk factors for re-revision.

Confounder	Univariable	*P* value	Multivariable	*P* value
Age at first revision	0.96 (0.95-0.97)	<.001	0.96 (0.94-0.98)	<.001
Gender		.03		.11
Female	1		1	
Male	1.45 (1.03-2.02)		1.32 (0.94-1.87)	
Indication for first revision				
Instability/dislocation	1.59 (1.13-2.23)	.07	2.47 (1.59-3.82)	<.001
Infection	1.44 (1.01-2.05)	.04	1.46 (0.78-2.38)	.19
Aseptic loosening	0.55 (0.29-1.04)	.07		
Fracture	0.49 (0.22-1.13)	.10		
Component dissociation	0.18 (0.03-1.32)	.09		
Unexplained pain	NA	NA		
Cuff insufficiency	NA	NA		
Impingement	NA	NA		
Glenoid implant wear	NA	NA		
Lysis humerus	NA	NA		
Lysis glenoid	NA	NA		
Stiffness	NA	NA		
Other/unspecified	0.82 (0.50-1.37)	.46		
Revision procedure			1.48 (0.88-2.51)	.14
Single-stage revision	0.72 (0.52-1.01)	.06		
Two-stage revision	1.67 (1.17-2.38)	<.001		
Modular exchange for indications other than infection	0.90 (0.51-1.60)	.73		
DAIR ± modular exchange for infection	0.79 (0.33-1.94)	.61		
Excision arthroplasty	0.50 (0.07-3.59)	.49		
Conversion to arthrodesis	NA	NA		
Revision implant				
Hemiarthroplasty	0.80 (0.41-1.57)	.51		
Reverse	1.04 (0.74-1.48)	.79		
Comorbidities (%)				
Previous myocardial infarction	0.98 (0.61-1.58)	.95		
Congestive cardiac failure	0.86 (0.49-1.52)	.61		
Peripheral vascular disease	1.25 (0.78-2.01)	.36		
Cerebrovascular disease	0.54 (0.32-0.93)	.03	0.58 (0.33-1.04)	.07
Dementia	0.83 (0.41-1.70)	.61		
Chronic pulmonary disease	1.10 (0.78-1.55)	.61		
Rheumatic disease	0.97 (0.64-1.46)	.88		
Peptic ulcer disease	0.34 (0.13-0.93)	.04	0.31 (0.11-0.84)	.02
Mild liver disease	1.20 (0.65-2.22)	.57		
Diabetes without complications	1.20 (0.82-1.76)	.35		
Diabetes with end organ damage	1.31 (0.64-2.67)	.46		
Hemi or paraplegia	1.29 (0.61-2.78)	.51		
Moderate or severe renal disease	0.41 (0.22-0.79)	.01	0.45 (0.22-0.92)	.03
Malignancy except skin	0.39 (0.20-0.74)	<.001	0.28 (0.19-0.77)	.01
Lymphoma	0.70 (0.10-5.00)	.72		
Leukaemia	1.12 (0.28-4.54)	.87		
Moderate or severe liver disease	1.65 (0.61-4.45)	.33		
Metastsis	0.48 (0.15-1.49)	.20		
HIV/AIDS	1	NA		
CCI	0.87 (0.81-0.94)	<.001	1.10 (1.00-1.23)	.05
Surgeon revision frequency	1.06 (1.01-1.10)	.01	1.05 (1.00-1.09)	.04

*DAIR*, débridement and implant retention; *CCI*, Charlson Comorbidity Index; *HIV*, human immunodeficiency virus; *AIDS*, acquired immune deficiency syndrome.

## Discussion

rTSA continues to gain popularity both in the UK and worldwide. With this increase in primary operations, an increase in revision case load is inevitable. When an rTSA is revised, this is commonly in a single-stage procedure (56.93%), and the revision implant is most commonly an rTSA (61.75%).

At 1 year, the incidence of re-revision is 15.91% (95% confidence interval [CI] 13.30-18.98); at 3 years, it is 21.41% (95% CI 18.34-24.92); and at 5 years, it is 23.18% (95% CI 19.90-26.91). These results concur with the earlier systematic review in Section 5.2, which found the estimated 1-year re-revision rate is 14% and 5-year re-revision rate of 23%. These re-revision rates show that once an rTSA has been revised, the risk is over 7 times higher for a second revision than the rate of primary rTSA revision with rates reported by the NJR of 1.68% at 1 year, 2.58% at 3 years, and 3.05% at 5 years.^15^ When infection was excluded as the primary revision cause the 1 year re-revision rate was 14.96 (95% CI 12.05-18.50) 3 year re-revision 19.15% (95% CI 15.82-23.08) and 5 Year of 21.04 (17.45-25.26) these results are similar to the Australian NJR study of aseptic second revisions which had a re-revision rate of 13% at 1 year, 18% at 3 years and 22% at 5 years.^10^ The mean follow-up period for the rTSAs who underwent primary revision was 3.82 years. With longer term follow-up the revision rates may in fact be higher than those shown in this study.

The revision procedures in the registry were performed by 244 consultants with a low mean caseload over the 10-year period. It is widely accepted that revision rTSA is a complex procedure and in primary arthroplasty it is established that surgeon volume of greater than 10.4 shoulder arthroplasties per annum is associated with better patient outcomes.^20^ While the regression modelling indicated an increased risk of re-revision with increasing surgeon volume, this finding should be interpreted with caution. It is possible that higher-volume surgeons are more likely to undertake more complex or technically demanding revision procedures, which may inherently carry a higher risk of failure. In addition, the analysis may be influenced by unmeasured confounding factors, such as the severity of underlying pathology or the complexity of cases referred to experienced centers. Despite the observed re-revision rates, these findings may still support the ongoing discussion around centralizing revision arthroplasty services to higher-volume centers, as such centers have been associated with improved patient outcomes and are currently being recommended for elbow arthroplasty in the UK.^1^^,^^6^^,^^9^^,^^11^^,^^20^

This study is the first to look at predictive factors of re-revision surgery in rTSA. Those who went on to have a successful primary revision were significantly older than the re-revision cohort (*P* = <.001). A younger age at first revision was found to be an independent predictor of re-revision with each 1 year decrease in age conferring to a 4% increased risk of re-revision (hazard ratio [HR] 0.96; CI 0.95-0.97). This may be explained due to confounding by indication with aversity to completing a further operation in an older patient with a more accepting cohort of reducing function or that younger patients are more likely to put increased stress and wear through the replacement, leading to earlier failure and would be less accepting of a poorer function. Male gender is an established risk factor for primary revision surgery in primary shoulder replacement; and in this study, the re-revision cohort had a significantly higher proportion of males in the cohort; however, when accounting for other confounders, it was not an independent predictor of re-revision.^7^^,^^18^^,^^22^

In terms of indication for primary revision, the analysis found if the primary revision was due to instability/dislocation this was an independent risk factor for re-revision with a 2-fold increased risk of re-revision compared to if the primary revision was done for any other indication (HR 2.47 [95% CI 1.59-3.82]). A study looking at failed revision rTSAs for instability vs. failed primary's for instability found that 27% of their patients who were revised for instability a second time had ongoing instability following revision compared to 7% in first revision, emphasizing the challenging nature of the complication to address.^13^ Instability is the leading cause of revision in rTSA, and there are numerous studies that aim to review risk factors for instability following primary rTSA, despite this, this study shows that given the increased risk of further revision when the primary revision diagnosis is instability, the underlying etiology may not be being sufficiently addressed in these patients in order to prevent further revision and should be a target for learning and outcome improvement.^5^^,^^12^^,^^13^

Infection was associated with an increased risk in univariable analysis however was not an independent risk factor for re-revision when accounting for other confounding factors (HR 1.46 [95% CI 0.78-2.38]). Those having a two-stage revision, which is most likely to be for infection, showed a trend towards an increased risk of re-revision; however, this was not statistically significant when accounting for other factors (HR 1.59 1.13-2.23 *P* = .07). A recent study reviewing outcomes of revision for infection vs. other revision indications have shown comparable results in terms of Patient Reported Outcome Measures (PROMs), revision, and reoperations.^23^ Indication for revision, however, is recorded at time of revision and therefore unexpected positive cultures are unlikely to be captured and this could also lead to under reporting of infection as an indication.

Comorbidities such as peptic ulcer disease, moderate or severe renal disease or malignancy except skin all inferred a reduced risk of re-revision which is likely to be attributable to confounding by indication. Those with severe comorbidities are less likely to be deemed suitable for an anesthetic for what would be a second revision procedure and therefore may be treated conservatively. This differs from primary revision risk factors where comorbidities such as diabetes and congestive cardiac failure have been shown to increase primary revision risk.^18^

The limitations of this study surround the data mining from the HES and NJR database. NJR records first revision however re-revision was identified using HES codes and therefore relies on accurate coding. Despite this, the re-revision rates detected are high and therefore should be used as a baseline assumption with possible higher rates. Unfortunately, the reason for second revision is unable to be identified from the HES or NJR data and this would be a useful metric to identify the leading cause of ongoing issues with the rTSA implant. In the NJR, data collection form DAIR without modular exchange was only captured from MDSv7 in 2018. Despite the NJR always deeming this as a revision procedure, there was concern prior to its inclusion in the form it may have been under-reported and therefore the numbers of DAIR with implant retention in this study may be lower than expected.^16^ Re-revision is an indicator of success explored in this study; however, it does not address patient function. PROMs are not routinely collected for revision procedures in the NJR. Given the high rate of re-revision with possible further failing, implants in terms of function that are not revised due to demographic factors, collection of revision PROMs should be considered to gauge the true picture of implant success. Despite these limitations, this is the largest study to look at predictors of re-revision in rTSA and emphasizes high revision rates when an rTSA is not primarily successful.

## Conclusion

Re-revision rates following revision rTSA are high with independent risk factors for failure being younger age and instability as primary revision diagnosis. These findings underscore the importance of careful patient selection and surgical planning. Centralizing revision procedures to high-volume centers may warrant consideration in health-care systems that support it, as this approach could enhance surgical expertise, optimize outcomes, and better address the challenges associated with managing failed primary rTSAs.

## Acknowledgment

We thank the NJR research committee and staff at the NJR, for facilitating this work. The authors have conformed to the NJR's standard protocol for data access and publication. The views expressed represent those of the authors and do not necessarily reflect those of the NJR steering committee, research subcommittee, or HQIP.

## Disclaimers:

Funding: The authors disclose receipt of the following financial or material support for the research, authorship, and/or publication of this article: an institutional British Elbow and Shoulder Society pump primer research grant and a Royal College of Surgeons Research Fellowship funded by the Arthritis Research Trust with support from the Rosetrees Trust.

Conflicts of interest: Olivia O'Malley is an RCS England Research Fellow. A. Rangan reports an institutional grant from NIHR and AO UK&I, as well as research and educational grants from DePuy J&J Ltd., unrelated to this study. He is also an Elected Trustee of the British Orthopaedic Association and is on the funding committee of NIHR i4i. The other authors, their immediate families, and any research foundation with which they are affiliated have not received any financial payments or other benefits from any commercial entity related to the subject of this article.

## Supplementary Data

Supplementary Appendixes S1-S4
