# Supplementary material for: Re-revision following revision of a failed primary reverse total shoulder arthroplasty: an analysis of the National Joint Registry and Hospital Episode Statistics for England
Source: JSES Int. 2025 May 22;9(5):1608–15. doi: 10.1016/j.jseint.2025.04.030 (PMC12490553; doi:10.1016/j.jseint.2025.04.030)
Supplement: Supplementary Appendixes S1-S4 [file mmc1.docx]

Appendix

Appendix 1

Where there were multiple indications listed, those with OA only as an indication were coded as Primary Osteoarthritis and osteoarthritis removed from other diagnosis codes. Primary Osteoarthritis was further split into those with and without an intact rotator cuff.

Appendix 2

| **Revision** | **OPCS-4.9 Code** | **Anatomy Code** | **Revision** | **OPCS-4.9 Code** |
| --- | --- | --- | --- | --- |
| **Other /Not Specified** | W430 | Z814 | **RSA** | O370 |
|  | W432 | Z891 |  | O371 |
|  | W433 | Z814 |  | O372 |
|  | W434 |  |  | O378 |
|  | W438 |  |  | O379 |
|  | W439 |  |  | O380 |
|  | W440 |  |  | O381 |
|  | W442 |  |  | O382 |
|  | W443 |  |  | O388 |
|  | W444 |  |  | O389 |
|  | W448 |  |  | O390 |
|  | W449 |  |  | O391 |
|  | W450 |  |  | O392 |
|  | W452 |  |  | O393 |
|  | W453 |  |  | O398 |
|  | W454 |  |  | O399 |
|  | W455 |  |  | O400 |
|  | W458 |  |  | O402 |
|  | W459 |  |  | O403 |
|  | W522 |  |  | O404 |
|  | W523 |  |  | O405 |
|  | W528 |  |  | O406 |
|  | W529 |  |  | O408 |
|  | W530 |  |  | O409 |
|  | W532 |  |  | W966 |
|  | W533 |  |  | W976 |
|  | W538 |  |  | W987 |
|  | W539 |  | **Hybrid** | O060 |
|  | W540 |  |  | O062 |
|  | W542 |  |  | O063 |
|  | W543 |  |  | O064 |
|  | W544 |  |  | O068 |
|  | W548 |  |  | O069 |
|  | W549 |  |  | O070 |
|  | W580 |  |  | O072 |
|  | W582 |  |  | O073 |
|  | W570 |  |  | O074 |
|  | W572 |  |  | O078 |
|  | W573 |  |  | O079 |
|  | W574 |  |  | O080 |
|  | W578 |  |  | O082 |
|  | W579 |  |  | O083 |
|  | W60 |  |  | O084 |
|  | W61 |  |  | O085 |
|  | W62 |  |  | O088 |
|  | W63 |  |  | O089 |
|  | W64 |  | **TSA** | W960 |
|  | W817 |  |  | W962 |
|  | W580 |  |  | W963 |
|  | Y037 |  |  | W964 |
| **HA** | W490 |  |  | W968 |
|  | W492 |  |  | W969 |
|  | W493 |  |  | W970 |
|  | W498 |  |  | W972 |
|  | W499 |  |  | W973 |
|  | W500 |  |  | W974 |
|  | W502 |  |  | W978 |
|  | W503 |  |  | W979 |
|  | W508 |  |  | W980 |
|  | W509 |  |  | W982 |
|  | W510 |  |  | W983 |
|  | W512 |  |  | W984 |
|  | W513 |  |  | W985 |
|  | W514 |  |  | W988 |
|  | W518 |  |  | W989 |
|  | W519 |  |  |  |

Appendix 3

**Hierarchy of revision indications**

1. Infection
2. Instability / dislocation
3. Aseptic loosening
4. Stiffness
5. Component dislocation
6. Glenoid implant wear
7. Glenoid erosion
8. Impingement
9. Lysis humerus
10. Lysis glenoid
11. Periprosthetic fracture
12. Unexplained pain
13. Cuff Insufficiency

Appendix 4

| **Comorbidity** | **ICD – 10 code** |
| --- | --- |
| **Myocardial Infarction** | I21, I22, I252 |
| **Congestive Cardiac Failure (CCF)** | I099, I110, I130, I132, I255, I420, I425-I429, I43, I50, P290 |
| **Peripheral Vascular Disease (PVD)** | I70, I71, I73.1, I73.8, I73.9, I77.1, I79.0, I79.2, K55.1, K55.8, K55.9, Z95.8, Z95.9 |
| **Cerebrovascular Disease (CVD)** | G45, G46, H34, I60-I69 |
| **Dementia** | F00-F03, F051, G30, G311 |
| **Chronic Pulmonary Disease (CPD)** | I278, I279, J40-J47, J60-J67, J684, J701, J703 |
| **Rheumatic disease** | M05, M06, M120, M315, M32-M34, M351, M353, M360 |
| **Peptic Ulcer Disease (PUD)** | K25-K28 |
| **Mild Liver Disease (MLD)** | B18, K700-K703, K709 , K713 - K715, K717, K73, K74, K752, K754, K758, K759, K760- K764, K768, K769, Z944 |
| **Diabetes without chronic complicationsstc** | E100, E101, E109, E110, E111, E119, E120, E121, E129, E130, E131, E139, E140, E141, E149 |
| **Diabetes with end organ damage** | E102- E105, E107, E112- E115, E117, E122-E125, E127, E132- E135, E137, E142- E145, E147, N083 |
| **Hemi or paraplegia** | G041, G114, G800, G801, G802, G81, G82, G830, G839 |
| **Moderate or severe renal disease** | I120, I131, I132, N03 , N012 -N017, N032-N037, N042-N047, N052-N057, N072-N077, N183-N185, N19, N250, N280, N290, Z490-Z492, Z992 |
| **Malignancy except skin** | C00–C26, C30–C34, C37–C41, C43, C45–C58, C60–C76, |
| **Lymphoma** | C81-C88, C90 |
| **Leukemia** | C91-C96 |
| **Moderate or Severe liver disease** | I85, I864, I982, I983, K704, K711, K713, K721, K729, K762, K763, K765-K767 |
| **Mets** | C77-C80 |
| **HIV/Aids** | B20-B24 |
